# The role of atropine in myopia control: insights into choroidal and scleral mechanisms

**DOI:** 10.3389/fphar.2025.1509196

**Published:** 2025-03-20

**Authors:** Longxiang Huang, Jingjin Zhang, Youfang Luo

**Affiliations:** ^1^ Department of Ophthalmology, The First Affiliated Hospital of Fujian Medical University, Fuzhou, China; ^2^ Department of Ophthalmology, National Regional Medical Center, Binhai Campus of the First Affiliated Hospital, Fujian Medical University, Fuzhou, China; ^3^ Fujian Institute of Ophthalmology, The First Affiliated Hospital of Fujian Medical University, Fuzhou, China; ^4^ Fujian Provincial Clinical Medical Research Center of Eye Diseases and Optometry, The First Affiliated Hospital of Fujian Medical University, Fuzhou, China; ^5^ Department of Rehabilitation, Fuzhou Second General Hospital, Fuzhou, China

**Keywords:** atropine, myopia, choroidal microcirculation, scleral hypoxia, extracellular matrix remodeling

## Abstract

In this study, we investigate the inhibitory effects of atropine on the progression of experimental myopia by targeting the functions of the choroid and sclera and exploring its potential therapeutic mechanisms. Form deprivation myopia (FDM) was induced in C57BL/6 mice, with treatment groups receiving atropine. We assessed the effects on ocular morphology, extracellular matrix (ECM) protein expression, choroidal and scleral thickness, and choroidal vascular index (CVI) through histopathology, immunofluorescence, and quantitative quantitative polymerase chain reaction (qPCR). *In vitro*, mouse scleral fibroblasts (MSFs) were treated with Na_2_S_2_O_4_ to induce hypoxia, followed by atropine treatment. Atropine treatment significantly reduced axial elongation and ECM remodeling in FDM mice, as indicated by a decrease in collagen volume fraction. It restored choroidal and scleral thickness and increased CVI, suggesting improved microcirculation. Atropine also modulated ECM protein expression and reduced the hypoxia marker Hypoxia-Inducible Factor-1α (HIF-1α). *In vitro*, atropine protected MSFs from hypoxia-induced damage, preserved cytoskeletal integrity, and modulated key signaling pathways, including P53 and β-catenin. These findings suggest that atropine holds promise for controlling myopia progression by improving choroidal microcirculation, reducing scleral hypoxia, and regulating ECM remodeling, supporting its therapeutic application in myopia management.

## 1 Introduction

The onset and progression of myopia lead to axial elongation and a series of ocular changes, including thinning of the sclera and choroid, elongation of the retina, optic disc tilt, parapapillary choroidal cavitation, and Bruch’s membrane defect (Jonas et al., 2023). Axial elongation is the most significant change and cause of myopia, a normal physiological phenomenon typically associated with height growth. The younger the child, the faster the axial elongation; after the age of 18, the axial length tends to stabilize (Xiang et al., 2012). Thinning of the sclera and choroid is another important phenotype of myopia, with scleral thinning primarily occurring posterior to the equator, most notably at the posterior pole (Jonas et al., 2023; Wu et al., 2021).

In recent years, significant breakthroughs have been made in understanding the pathogenesis of myopia. Myopia results from excessive axial elongation and reduced scleral strength and thickness caused by extracellular matrix (ECM) remodeling (Wu et al., 2018). The signaling pathways potentially involved in this process include the dopamine pathway, Wnt signaling pathway, BMP-2 pathway, and retinoic acid receptor pathway (Luo et al., 2017; Chen et al., 2017; Ma et al., 2014; Li et al., 2015; Wang et al., 2014). However, these studies do not fully explain how the signals that induce myopia progression are transmitted among different ocular tissues. The choroid plays a crucial role in this signal transmission. It is generally believed that scleral hypoxia is a key pathway through which myopia-related visual signals are transmitted from the retina to the sclera via the choroid. External visual stimuli may regulate choroidal blood flow, leading to scleral hypoxia and subsequent myopia (Wu et al., 2018; Zhao C. et al., 2020). In the scleral hypoxia pathway involved in myopia, Hypoxia-Inducible Factor-1α (HIF-1α) plays a critical role: hypoxic conditions increase HIF-1α expression, activating and upregulating factors such as P-eIF-2α and p-mTOR, resulting in fibroblast differentiation, an increased ratio of myofibroblasts to fibroblasts, ECM remodeling, reduced collagen production, and eventually axial myopia (Wu et al., 2018; Zhao F. et al., 2020).

Currently, drug intervention is at the forefront of scientific exploration in controlling myopia progression. The ATOM studies conducted by the Singapore National Eye Centre (SNEC) reported that using 0.01% atropine sulfate eye drops in children resulted in an average myopia progression of −1.38D over 5 years, with a 60% effectiveness rate compared to the control group, and fewer side effects and rebound phenomena than higher concentrations of atropine drops (Tong et al., 2009; Chia et al., 2016). Subsequent studies have confirmed the efficacy and safety of low-concentration atropine in controlling myopia (Yam et al., 2023; Ha et al., 2022). In Australia, 0.01% atropine (Eikance^®^) was approved in 2024 for slowing myopia progression in children aged 4–14 years, specifically when annual progression exceeds −1.0 D. Concurrently, the EMA is evaluating NVK002 (atropine sulfate) under a Paediatric Investigation Plan (PIP), with a Marketing Authorisation Application (MAA) validated in May 2024 for children aged 3–18 years (EMA/303040/2021, EMA/777575/2022, and EMA/CHMP/539348/2024). These advancements align with evidence from the ATOM studies, which demonstrated the efficacy and safety of low-dose atropine, particularly the 0.01% formulation, in reducing myopia progression and axial elongation with minimal rebound effects. However, the mechanism by which atropine controls myopia progression remains unclear (Upadhyay and Beuerman, 2020).

Pharmacologically, atropine is a reversible competitive antagonist that binds to all five subtypes of muscarinic receptors (MRs, MR1-MR5), suggesting that its protective effect against myopia is primarily mediated through MRs (Upadhyay and Beuerman, 2020). However, evidence specifically supporting atropine’s control of myopia through MR inhibition is insufficient. Previous studies suggested that high doses of MR antagonists might exert cytotoxic effects on scleral cells, thereby inhibiting the function of glycosaminoglycan-producing cells (McBrien et al., 2013). Further research showed that atropine could inhibit glycosaminoglycan synthesis in chick cartilaginous scleral cells even without any acetylcholine source, implying that atropine might also control myopia progression through non-MR mechanisms (Lind et al., 1998). Notably, MR inhibitors, such as atropine, have been shown to increase choroidal thickness, consistent with atropine’s ability to alleviate vascular spasm (Cristaldi et al., 2020). Studies found that the selective MR agonist carbachol upregulated HIF-1α expression in cardiomyocytes through the *PI3K*/*AKT*/*HIF-1α* pathway, an effect that atropine could block (Kakinuma et al., 2005). This characteristic of atropine suggests its potential role in regulating choroidal function and the scleral hypoxia pathway.

This study aims to determine whether atropine inhibits myopia progression by improving choroidal microcirculation and scleral hypoxia, inhibiting the HIF-1α signaling pathway, and targeting MRs on scleral fibroblasts to inhibit ECM production and remodeling. To this end, we employed two validated models: 1) a form deprivation myopia (FDM) mouse model, which reliably mimics human myopia through axial elongation and scleral thinning (Wu et al., 2018), and 2) an *in vitro* Na_2_S_2_O_4_-induced hypoxia model in mouse scleral fibroblasts (MSFs), a system proven to replicate hypoxia-driven HIF-1α activation and ECM dysregulation (Zhao et al., 2019). Both models were treated with atropine, and their morphological and molecular responses were analyzed to elucidate the pathways through which atropine inhibits myopia progression.

## 2 Materials and methods

### 2.1 Animals

Adult C57BL/6 mice (males, 3 weeks) were purchased from the Skbex Biotechnology (Henan Province, China). The temperature of the breeding environment was 23°C–25°C, the humidity was 50% ± 10%, and the photoperiod was 12/12 h. During the feeding period, the mice had *ad libitum* access to movement, food, and water. All procedures of animal studies were in accordance by institutional regulations and with the approval of the Laboratory Animal Welfare and Ethics Committee of Fujian Medical University [(2015)084-2].

### 2.2 FDM mouse model and atropine treatment

All mice with normal anterior segments as confirmed by slit-lamp microscopy had one randomly selected eye covered with a PVC hemisphere to induce Form deprivation myopia (FDM). The hemisphere was securely attached to the fur using adhesive. The other eye remained uncovered as a control. The PVC hemispheres were checked three times daily to ensure they remained attached, and if detached, were reattached promptly to limit visual exposure to under 8 h. All procedures were performed by a single trained technician to minimize variability.

Atropine (Cat. No. HY-B1205; MedChemExpress, NJ, United States) was dissolved in saline and further diluted to the working concentration (10 mg/mL), and was administered as eye drops, with the drug being delivered through a small hole in the PVC hemisphere for the covered eye, which was immediately sealed after administration. Atropine eye drops were administered to the uncovered eye through a small hole in the PVC hemisphere using a calibrated micropipette to deliver a precise volume (2 µL). The form deprivation lasted for 3 weeks. The atropine dosage of 10 mg/mL was selected based on prior preclinical studies (McBrien et al., 2013; Upadhyay and Beuerman, 2020) and pilot dose-response experiments, which demonstrated its efficacy in reducing axial elongation and ECM remodeling without inducing adverse effects. This concentration accounts for species-specific differences in drug metabolism and aligns with established practices in translational research (Cristaldi et al., 2020).

The experiment was divided into groups based on different treatments: 1) Mock group: uncovered eye treated with saline; 2) Atropine group: uncovered eye treated with 10 mg/mL atropine; 3) FDM group: covered eye treated with saline; 4) FDM + Atropine group: covered eye treated with 10 mg/mL atropine (*N* = 4 per group). Eye drops were administered three times daily, with the covered eye receiving treatment through a small hole in the PVC hemisphere, which was immediately closed after administration to minimize visual exposure. The sample size of four mice per group was determined based on a power analysis (GPower 3.1.9.7) to detect a large effect size (d = 2.1) with 80% power at α = 0.05, consistent with prior studies using the FDM model (Wu et al., 2018; Zhao C. et al., 2020). To assess the drug safety in the ophthalmic of 10 mg/mL atropine, anterior segments were observed every week by slit lamp biomicroscope (S350, Shanghai Medi Works Precision Instruments, Hangzhou, China).

### 2.3 Histopathologic examination

Mice were euthanized by cervical dislocation on day 21 after treatment as previously described and both eyes were enucleated immediately. Mark the corneal apex before fixation. Eyes were fixed in formaldehyde, acetic acid, and saline (FAS) fixative (Wuhan Servicebio, Wuhan, China) for 48 h, embedded in paraffin, and cut into serial 4 µm tissue sections. For each eye sample, sections near the corneal apex to the optic nerve plane were taken for hematoxylin and eosin (HE) staining to visualize cellular and tissue structure and Masson’s trichrome staining to assess collagen expression. Tissue from both sides of the disc was used for subsequent analysis. For each eye, two sections (one from each side of the optic nerve) were analyzed, resulting in eight measurements per group (*N* = 4 mice, *n* = 8 sections). Images were taken with a Leica DM4 microscope (DM400B; Leica, Wetzlar, Germany) and analyzed using ImageJ software. Measure the axial length, transverse diameter, and lens thickness of the eyes in each group of mice in the images of HE-stained sections. Collagen expression of the sclera was quantified by collagen volume fraction (CVF) and calculated as the ratio of blue-stained tissue area to total area (%). The assessment of choroidal structure was quantified by choroidal vascular index (CVI) based on corresponding binarized images processed with ImageJ software.

### 2.4 Cell culture and treatment Paradigms

Primary MSFs were isolated from postnatal day 10 mice. Eyes were enucleated using sterilized forceps and scissors and transferred to a dish containing cold PBS. Using a stereomicroscope, extraocular tissues and the cornea were removed, and the sclera was carefully dissected from the underlying uveal tract and retina. The scleral tissue was minced into small pieces (1 mm), transferred into a tube containing collagenase type I solution (1 mg/mL in HBSS), and incubated at 37°C for 1 h with gentle agitation to digest the extracellular matrix. The digested tissue was centrifuged at 300 g for 5 min to pellet the cells. The supernatant was discarded, and the pellet was resuspended in trypsin-EDTA (0.25%), incubated for 5 min at 37°C to further dissociate the cells, and then neutralized by adding an equal volume of culture media (DMEM with 10% FBS). The cell suspension was passed through a 70 μm cell strainer to remove undigested tissue fragments and centrifuged at 300 g for 5 min. The cell pellet was resuspended in fresh culture media, plated in a tissue culture flask or dish, and incubated at 37°C in a humidified incubator with 5% CO_2_. The culture media were changed every 2–3 days, and cell growth and confluence were monitored under a microscope. Cells were passaged when they reached 70%–80% confluence using trypsin-EDTA.

Cells in passages 3 to 6 were used in all experiments. The cells were treated with 1, 2, 5, 10 ng/mL Na_2_S_2_O_4_ (90%; MACKLIN, Shanghai, China) for 6 or 12 h, or treated with 3, 10, 30, 100, 300 μM Atropine for 24 h. The concentrations of Na_2_S_2_O_4_ (10 ng/mL) and atropine (100 μM) used in subsequent experiments were selected based on pilot dose-response studies. For Na_2_S_2_O_4_, 10 ng/mL reliably induced hypoxia markers (e.g., HIF-1α upregulation) while maintaining cell viability above 70%. For atropine, 100 μM effectively mitigated hypoxia-induced damage, preserved cytoskeletal integrity, and modulated key signaling pathways without cytotoxicity. These doses were chosen to balance efficacy and safety, aligning with prior studies (Zhao et al., 2019; Upadhyay and Beuerman, 2020).

MSFs were first treated with a medium containing 10 ng/mL Na_2_S_2_O_4_ for 6 h, and then the medium was replaced with one containing 100 μM atropine for an additional 24 h. The blank control group was set in all experiments, cells were treated with a culture medium only. All the drugs were further diluted in the cell culture medium to reach their working concentrations. The morphology images of MSFs were taken under a Leica DM4 microscope.

### 2.5 Cell viability analysis

Cell Counting Kit-8 (CCK-8) assay kit (Dojindo Molecular Technologies, Kumamoto, Japan) was used to detect cell viability. MSFs were seeded in 96-well plates at 1 × 10^4^ cells per well and treated with different concentrations Na_2_S_2_O_4_ for 6 h or atropine for 24 h, or treated with 10 ng/mL Na_2_S_2_O_4_ for 6 h and 100 μM atropine for 24 h in sequence. Three to six replicate wells were repeated for each group. The original culture medium was discarded and a new Opti-MEM reduced serum medium (31985062; ThermoFisher, NY, United States) containing 10 µL CCK-8 reagent was added to each well. After incubation for 3 h, the absorbance was measured at 490 nm.

### 2.6 Immunofluorescence analysis

Mouse eye paraffin sections were dewaxed by xylene and gradient concentrations of ethanol (*N* = 4 per group). After antigen repair by sodium citrate, the sections prepared as mentioned above were blocked with 10% BSA at RT for 1 h after and incubated at 4°C overnight with the anti-fibronectin (Fn; TA336854, rabbit polyclonal antibody, 1:200; OriGene Technologies Inc., Rockville, MD, United States) and anti-Col1a1 (Col1; TA374961, rabbit polyclonal antibody, 1:200; OriGene Technologies Inc., Rockville, MD, United States). Then, sections were incubated with Alexa Fluor 594 goat anti-rabbit (1:1000; Invitrogen, Carlsbad, CA, United States) and further counterstained with DAPI for 5 min and observed under a fluorescence microscope. The fluorescence intensity in the sclera and choroid area was quantified by using ImageJ.

MSFs were seeded in round cover slips to obtain cell climbing slices. After treatment, cell climbing slices were fixed with 4% PFA at RT for 10 min and further incubated with 5% BSA and 0.5% Triton X-100 at RT for 1 h (*n* = 4 per group). For immunofluorescence, cells were incubated at 4°C overnight with the primary antibodies against FN (ab45688, rabbit monoclonal antibody, 1:200; Abcam, Cambridge, MA, United States), α-SMA (ab108424, rabbit monoclonal antibody, 1:100; Abcam, Cambridge, MA, United States), COL4A3 (COL4; ab111742, rabbit polyclonal antibody, 1:250; Abcam, Cambridge, MA, United States), and COX-4 (ab16056, rabbit polyclonal antibody, 1:200; Abcam, Cambridge, MA, United States) followed by incubation with Alexa Fluor 488/594 goat anti-rabbit (1:1000; Invitrogen, Carlsbad, CA, United States). For F-actin staining, cells were incubated with Rhodamine-phalloidin (PHDR1, 1:1000; Cytoskeleton, Denver, CO, United States) at room temperature for 30 min. Subsequently, the cell climbing slices were taken out and counterstained with DAPI (F6057-20ML, Sigma, MO, United States) for 5 min, and observed under a fluorescence microscope. The results were quantified by ImageJ as relative fluorescence intensity.

### 2.7 Quantitative polymerase chain reaction (qPCR)

After FDM-induced and treatment by 10 mg/mL atropine as previously described, sclera tissues were cut from the eye. Total RNA was extracted from TM tissue and cultured HTMCs with Trizol Reagent (Invitrogen, CA, United States). The absorbance values of RNA at OD 260 were detected using a spectrophotometer (Biophotometer plus, Eppendorf, Germany) to calculate the RNA content, and reverse transcription reactions were conducted using a kit (RevertAid First Strand cDNA Synthesis Kit; Fermentas, Thermo Fisher Scientific, Pittsburgh, PA, United States). SYBR Green Real-Time PCR Master Mix (Toyobo, Osaka, Japan) was used for quantitative real-time PCR (qPCR) on a Rotor-Gene Q cycler (QIAGEN, Germantown, MD, United States). Each reaction was run in triplicate. Transcript abundance was reported as relative to *GAPDH* levels and was calculated using the 2^−ΔΔCt^ method. qPCR was performed in three technical replicates on each biological replicate. Primer pairs used for PCR amplification in this study were provided as followed: *Fn* sense, ATG​TGG​ACC​CCT​CCT​GAT​AGT; antisense, GCC​CAG​TGA​TTT​CAG​CAA​AGG; *Hif-1α* sense, ACC​TTC​ATC​GGA​AAC​TCC​AAA​G; antisense, CTG​TTA​GGC​TGG​GAA​AAG​TTA​GG; GAPDH sense TGA​CCT​CAA​CTA​CAT​GGT​CTA​CA; and antisense, CTT​CCC​ATT​CTC​GGC​CTT​G.

### 2.8 Western blot

MSFs seeded in two 12-well plates were collected after being treated with 10 ng/mL Na_2_S_2_O_4_ for 6 h and 100 μM atropine for 24 h in sequence. Total proteins were extracted from MSFs using radioimmunoprecipitation assay (RIPA) lysis buffer (CWBio, Beijing, China) containing a 1% protease inhibitor cocktail (Sigma-Aldrich). After incubation on ice for 30 min, debris was removed by centrifugation (13,000 rpm) at 4°C and protein concentration was quantified by BCA assay. Proteins were diluted in 5× loading buffer and denatured at 98°C for 3 min 20 μg protein samples were fractionated by SDS-PAGE using the commercial polyacrylamide gel (Bio-Rad, Hercules, CA, United States) and transferred onto a polyvinylidene fluoride membrane (Millipore, Burlington, MA, United States). Membranes were blocked in 5% nonfat dried milk in 1× Tris-buffered saline with 0.1% Tween-20 (TBS-T) at room temperature for 1 h. Subsequently, membranes were incubated with the indicated primary antibodies against phosphorylated endothelial NO synthase (p-eNOs, phospho T495; 9574, rabbit monoclonal antibody, 1:1000; Cell Signaling Technology, Danvers, MA, United States), β-catenin (ab32572, rabbit monoclonal antibody, 1:1000; Abcam, Cambridge, MA, United States), ZO1 (5406, rabbit monoclonal antibody, 1:1000; Cell Signaling Technology, Danvers, MA, United States), P53 (9282, rabbit monoclonal antibody, 1:1000; Cell Signaling Technology, Danvers, MA, United States), and GAPDH (AF5718, goat polyclonal antibody, 1 mg/mL; R&D Systems, Minneapolis, MN, United States) overnight at 4°C. After washing with 1 × TBS-T for 30 min, membranes were incubated with corresponding secondary antibodies (1:10000; ZSGB-Bio) at room temperature for 1 h. Bands were visualized with a Fluor ChemE (ProteinSimple) and ImageJ was used to analyze the gray value of each protein band.

### 2.9 Protein-protein interaction (PPI) network visualization

The PPI network was constructed to explore the molecular interactions relevant to myopia and the response to atropine treatment. First, gene lists associated with myopia-related pathways, including ECM remodeling, choroidal blood flow regulation, and cellular hypoxia responses, were submitted to the STRING website (https://cn.string-db.org/). The analysis was conducted using a medium confidence score threshold (0.4) to identify potential interactions among the selected genes. After obtaining the PPI data from STRING, the interaction network was imported into Cytoscape software (version 3.9.1) for visualization and further analysis. In Cytoscape, nodes representing proteins were color-coded according to their functional categories: MR subtypes (blue), key signaling molecules and ECM components (yellow), and other proteins involved in choroidal and scleral function (green). The edges between nodes represent predicted associations, which include direct physical interactions as well as indirect functional interactions, such as co-expression and pathway co-occurrence.

### 2.10 Statistics

All data were analyzed using SPSS 29 software (IBM-SPSS 29. 0. 0. 0; Chicago, IL, United States). One-way ANOVA was used for comparison among four or more groups in this study, followed by an LSD *post hoc* test for homogeneous variances or a Games-Howell *post hoc* test for non-homogeneous variances. A paired t-test was used to analyze the cell viability of MSFs after 6 h and 12 h of Na_2_S_2_O_4_ induction. Pearson correlation analysis and linear regression models were applied to examine the association between axial length and choroidal thickness, scleral thickness, and CVI. A value of *P* < 0.05 was considered statistically significant. Variation is expressed as SEM.

## 3 Results

### 3.1 Atropine reduces axial length in FDM mouse models

**FIGURE 1:**
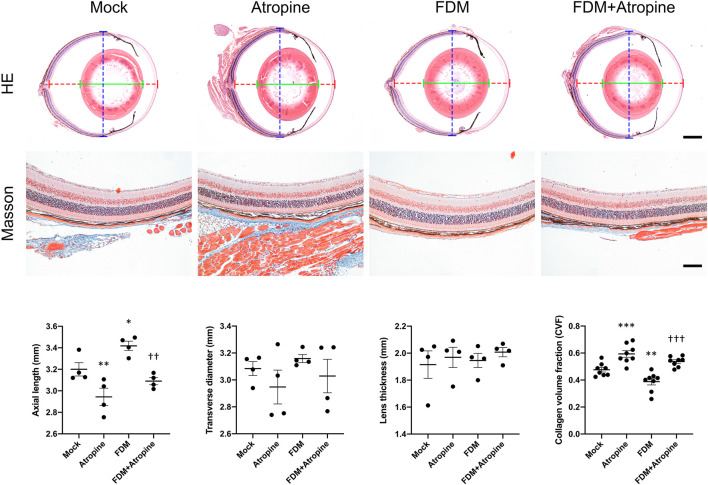
Effects of Atropine on Ocular Morphology in FDM Mouse Models. Representative HE stained images and quantitative analysis of axial length (dotted red line), transverse diameter (dotted blue line), and lens thickness (green line) of ocular cross-sections from mock, atropine-treated, FDM, and FDM + atropine-treated mice (*N* = 4 per group). Masson’s trichrome stained images showing collagen distribution and structure in the sclera of the different treatment groups. Collagen volume fraction (CVF) measured in the sclera of the different treatment groups (*N* = 8 per group). Data are presented as mean ± SEM. ^*^ *P* < 0.05, ^**^ *P* < 0.01, ^***^ *P* < 0.001 compared to mock; ^††^ *P* < 0.01, ^†††^ *P* < 0.001 compared to FDM. Scale bar: 5 mm in HE staining and 100 μm in Masson’s staining.

We investigated the effects of atropine on ocular morphology and collagen volume fraction in FDM mouse models. HE stained images revealed significant changes in the axial elongation of the treated groups. Atropine treatment markedly reduced axial elongation in FDM mice, indicating its potential in mitigating myopia progression. Additionally, Masson’s trichrome staining provided insights into the ECM remodeling. The reduction in CVF in the sclera of atropine-treated FDM mice compared to FDM alone suggests that atropine effectively inhibits the ECM remodeling processes associated with myopia progression. Quantitative analysis further confirmed that atropine significantly reduced both axial length and CVF, highlighting its therapeutic potential in managing myopia by targeting structural changes in the eye (Figure 1).

### 3.2 Atropine increase choroidal and scleral thickness and CVI in FDM mouse models

**FIGURE 2:**
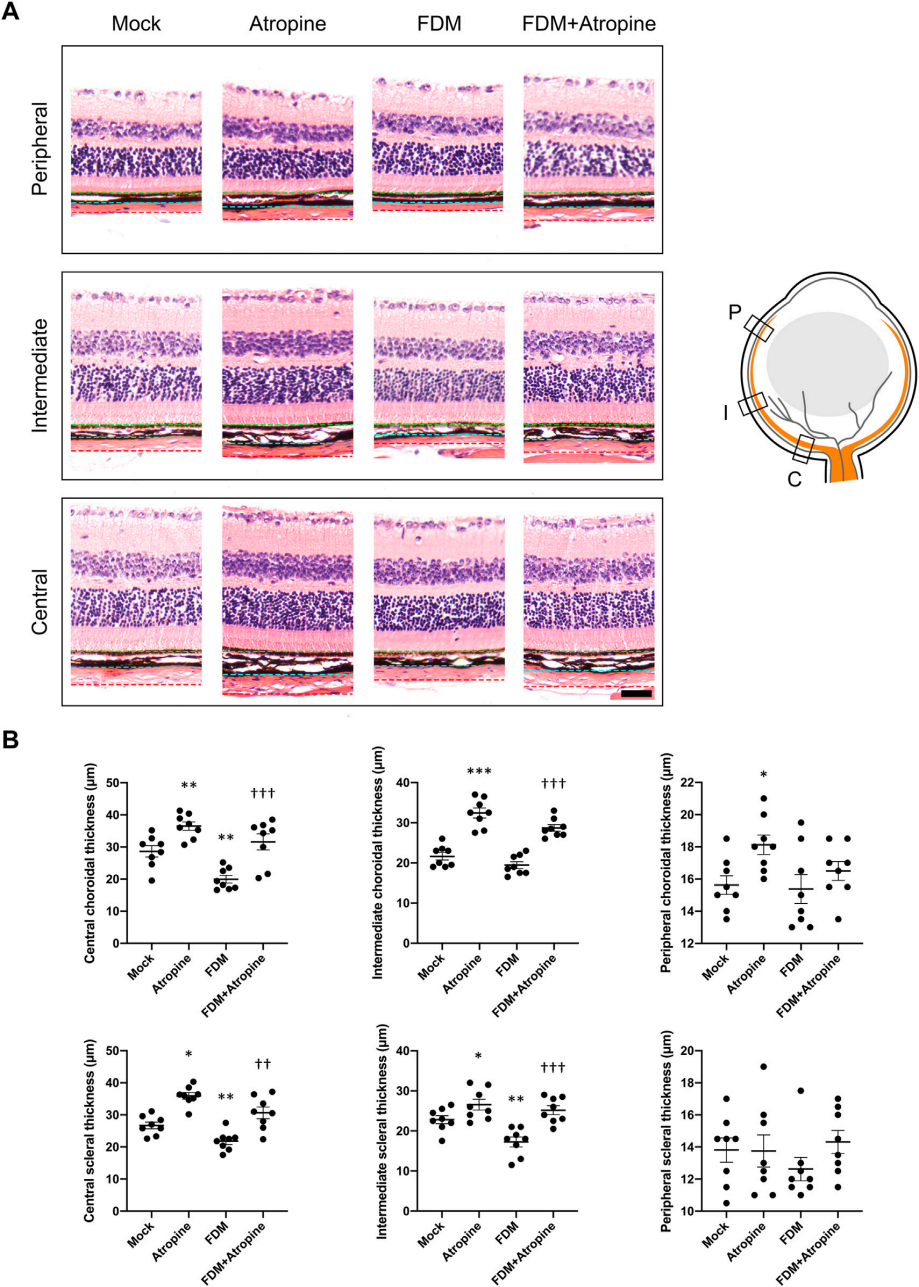
Effects of Atropine on Choroidal and Scleral Thickness in Different Regions of the Eye in FDM Mouse Models. **(A)** Representative HE stained images showing the peripheral, intermediate, and central regions of the choroid and sclera from mock, atropine-treated, FDM, and FDM + atropine-treated mice. The green dotted line indicates the outer boundary of the sclera, the blue dotted line marks the boundary between the sclera and choroid, and the red dotted line denotes the boundary between the choroid and retina. The schematic diagram on the right illustrates the locations of the peripheral (P), intermediate (I), and central (C) regions in the eye. **(B)** Quantitative analysis of choroidal and scleral thickness (μm) in the central, intermediate, and peripheral regions across the four groups, analyzed 8 images per group (*n* = 8 per group). The data show significant differences in thickness between the groups. Data are presented as mean ± SEM. ^*^ *P* < 0.05, ^**^ *P* < 0.01, ^***^ *P* < 0.001 compared to mock; ^†^ *P* < 0.05, ^††^ *P* < 0.01, ^†††^ *P* < 0.001 compared to FDM. Scale bar: 50 μm.

The effects of atropine on choroidal and scleral thickness were assessed in various regions of the eye using HE staining. In the central, intermediate, and peripheral regions, atropine treatment was observed to restore both choroidal and scleral thickness in FDM mice, suggesting that atropine can counteract the thinning effects induced by myopia (Figure 2A). This restoration of thickness in the sclera and choroid is critical as these structures play a significant role in maintaining the structural integrity and function of the eye. The quantitative analysis demonstrated the beneficial effects of atropine across all examined regions, indicating its broad impact on ocular tissue remodeling (Figure 2B).

**FIGURE 3:**
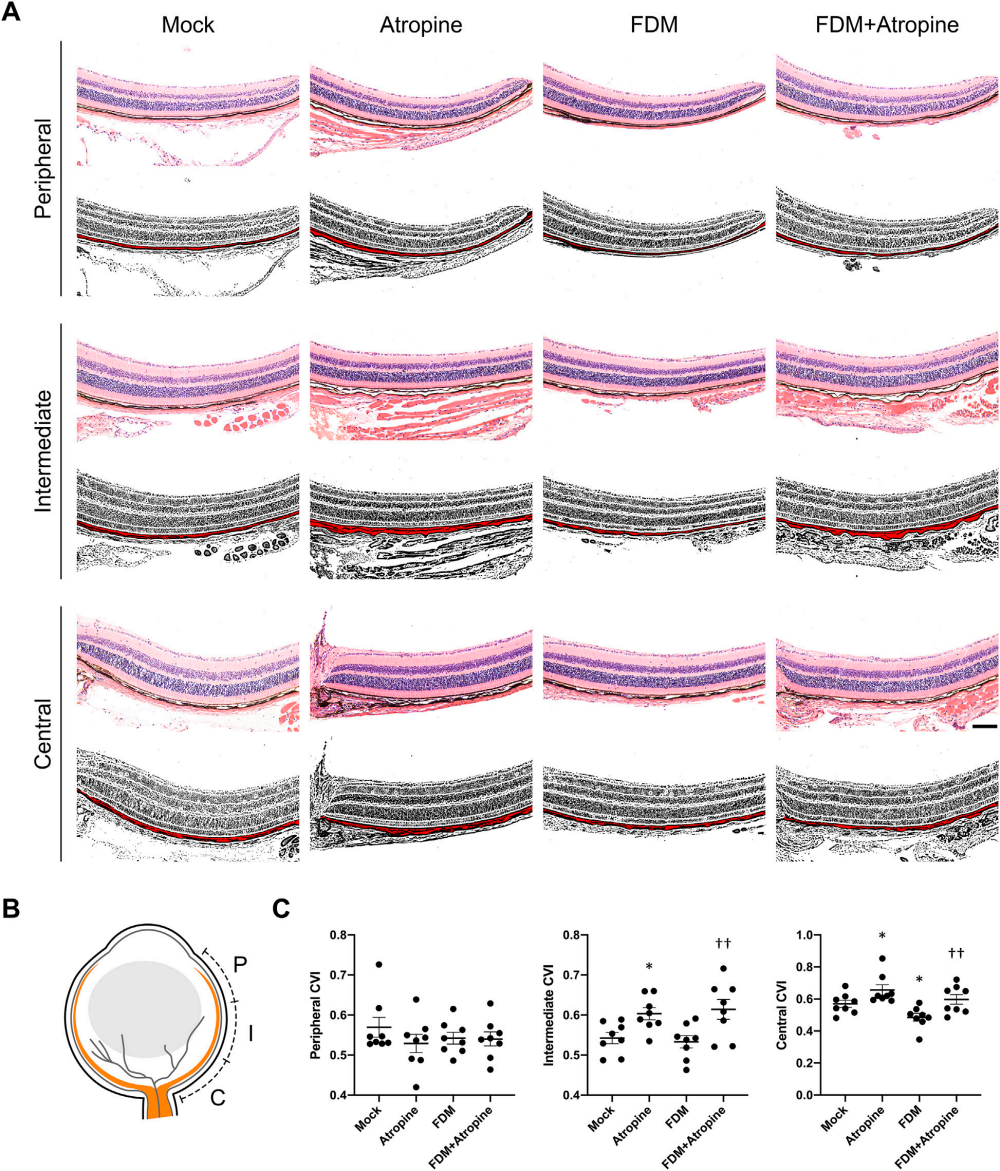
Effects of Atropine on CVI in Different Regions of the Eye in FDM Mouse Models. **(A)** Representative HE stained images and corresponding binarized images processed with ImageJ software, showing the peripheral, intermediate, and central regions of the choroid from mock, atropine-treated, FDM, and FDM + atropine-treated mice. The red areas in the binarized images indicate the choroidal vascular area. Scale bar: 200 μm. **(B)** Schematic diagram showing the locations of the peripheral (P), intermediate (I), and central (C) regions in the eye. **(C)** Quantitative analysis of the choroidal vascular index (CVI) in the peripheral, intermediate, and central regions across the four groups, analyzed 8 images per group (*n* = 8 per group). Data are presented as mean ± SEM. ^*^ *P* < 0.05 compared to mock; ^††^ *P* < 0.01 compared to FDM.

To further explore atropine’s role, we examined the CVI, a critical parameter reflecting vascular health and function (Ferrara et al., 2021). Using immunofluorescence and ImageJ software for image analysis, we observed that atropine treatment significantly increased CVI in the central and intermediate regions of the choroid in FDM mice (Figures 3A, B). The enhancement in CVI suggests that atropine improves choroidal blood flow and vascular health, which may be a mechanism through which it exerts its protective effects against myopia. The quantitative analysis supported these findings, indicating that atropine enhances vascular indices across all examined regions, potentially alleviating the hypoxia-induced changes seen in myopia (Figure 3C).

**FIGURE 4:**
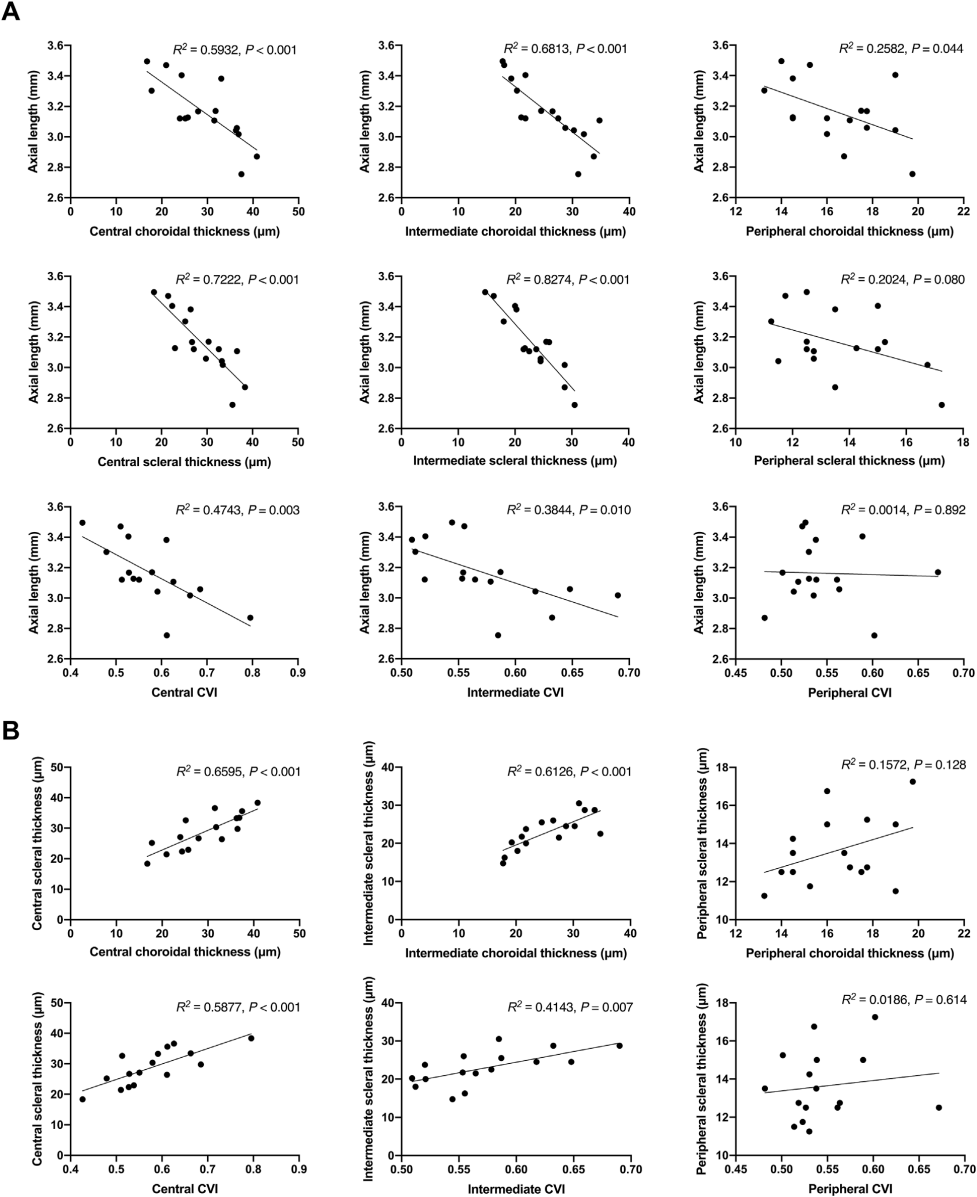
Correlation Analysis of Axial Length with Choroidal and Scleral Thickness, and CVI in FDM Mouse Models. **(A)** Scatter plots showing the correlation between axial length and choroidal thickness in the central, intermediate, and peripheral regions, scleral thickness in the central, intermediate, and peripheral regions, and CVI in the central, intermediate, and peripheral regions. The *R* ^*2*^ values and *P*-values indicate the strength and significance of the correlations. **(B)** Scatter plots showing the correlation between choroidal thickness and scleral thickness in the central, intermediate, and peripheral regions, and the correlation between CVI and scleral thickness in the central, intermediate, and peripheral regions. The *R* ^*2*^ values and *P*-values indicate the strength and significance of the correlations.

Correlation analysis provided further insights into the relationships between axial length, choroidal and scleral thickness, and CVI. Strong negative correlations were found between axial length and both choroidal and scleral thickness in the central and intermediate regions, indicating that increases in tissue thickness are associated with shorter axial lengths (Figure 4A). Additionally, positive correlations between choroidal thickness and scleral thickness, as well as between CVI and scleral thickness, were observed, suggesting interconnected growth and health of these tissues (Figure 4B). These correlations emphasize the multifaceted role of atropine in modulating various ocular parameters to control myopia progression.

### 3.3 Atropine modulates ECM protein expression in FDM mouse models

**FIGURE 5:**
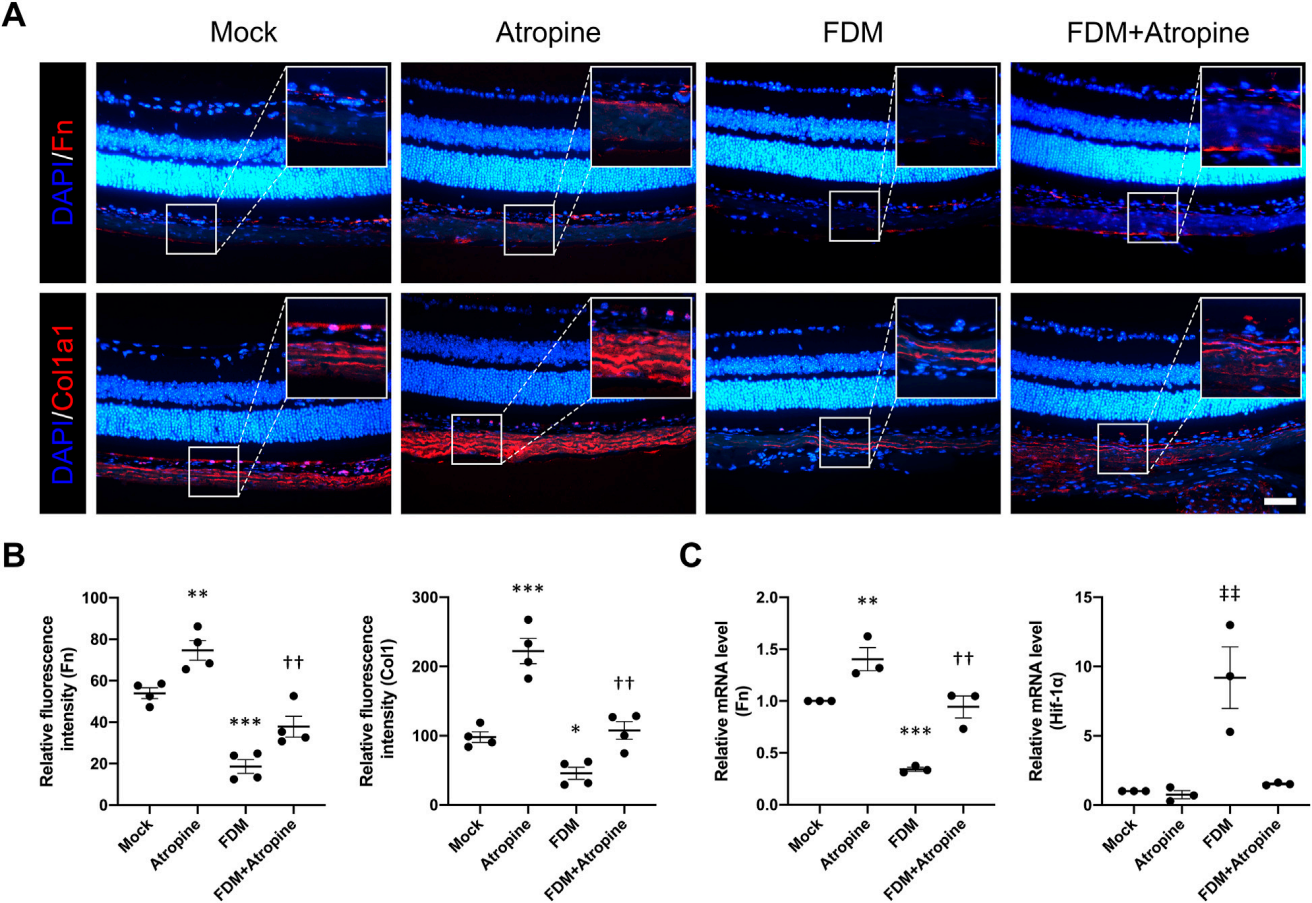
Effects of Atropine on ECM Protein Expression and mRNA Levels in FDM Mouse Models. **(A)** Representative immunofluorescence images showing the expression of Fn (red) and Col1a1 (red) in the sclera of mock, atropine-treated, FDM, and FDM + atropine-treated mice. DAPI (blue) was used to stain cell nuclei. Insets show higher magnification of the selected areas. Scale bar: 50 μm. **(B)** Quantitative analysis of relative fluorescence intensity of Fn and Col1a1 in the sclera from the four groups, analyzed 4 images per group (*n* = 4 per group). **(C)** Relative mRNA levels of Fn and Hif-1α in the sclera, as measured by qPCR, from the four groups. The expression of each mRNA level was relative to *GAPDH* and was normalized to the mock group (*n* = 3 per group). Data are presented as mean ± SEM. **P < 0.01, ^***^ *P* < 0.001 compared to mock; ^††^ *P* < 0.01 compared to FDM; ^‡‡^ *P* < 0.01 compared to Atropine.

We examined the expression of ECM proteins, including Fn and Col1a1, in the sclera of FDM mice. Immunofluorescence staining revealed that FDM induced significant decreases in the expression of Fn and Col1a1 (Figure 5A). However, atropine treatment significantly induced the expression levels of these ECM proteins in FDM mice. The quantitative analysis of relative fluorescence intensity further confirmed these observations, showing a substantial induction in ECM protein levels with atropine treatment (Figure 5B). Additionally, qPCR analysis demonstrated that atropine increased the mRNA levels of Fn and decreased the Hif-1α in FDM mice, indicating its regulatory effect on ECM gene expression and hypoxia-related pathways (Figure 5C). These findings suggest that atropine exerts its anti-myopia effects, at least in part, by modulating ECM remodeling and hypoxia signaling pathways in the sclera.

### 3.4 Atropine protects MSFs from Na_2_S_2_O_4_-Induced hypoxia

**FIGURE 6:**
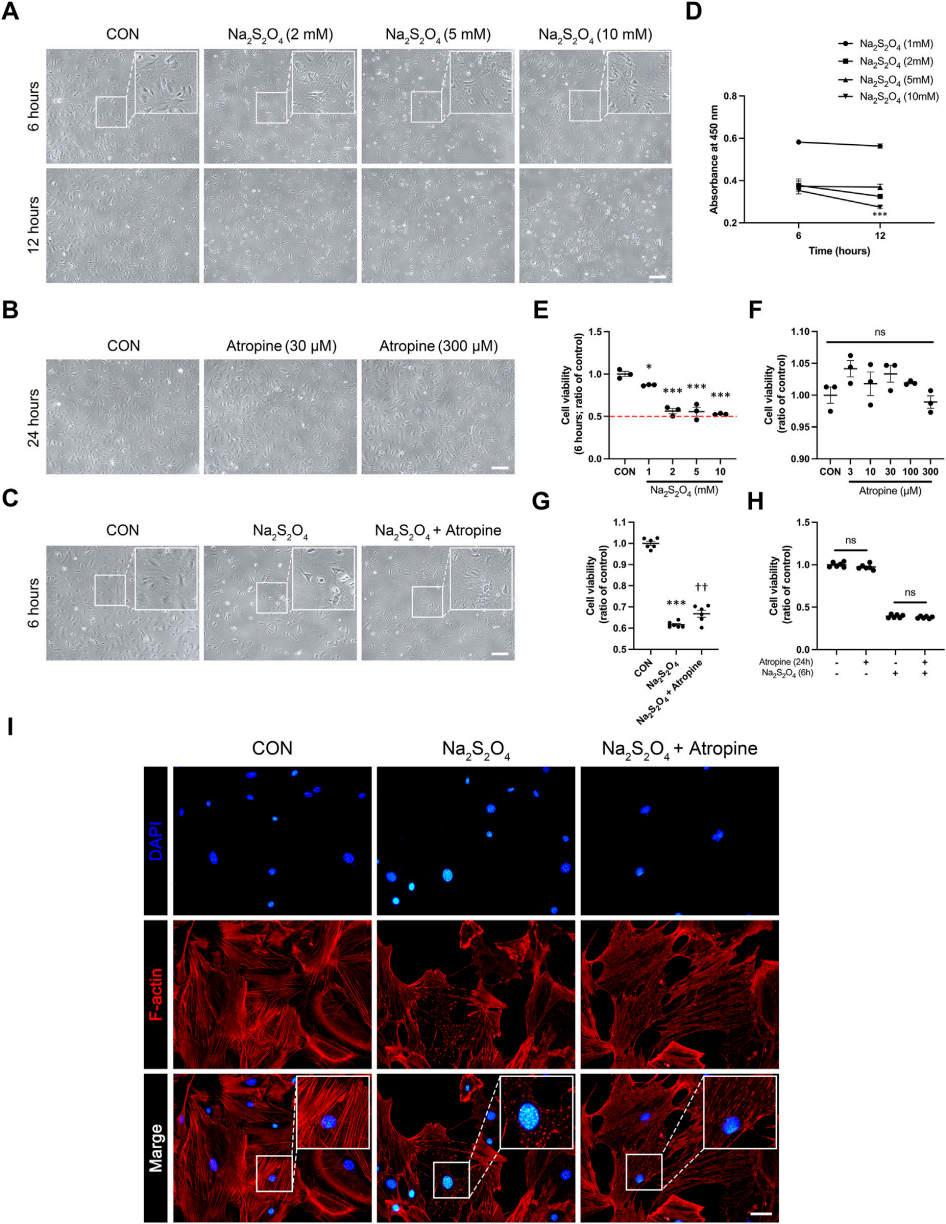
Effects of Atropine on Cell Viability and Cytoskeleton Integrity in Na_2_S_2_O_4_-Induced Hypoxia Model in MSFs. **(A)** Representative phase-contrast images showing the morphology of MSFs after 6 and 12 h of treatment with different concentrations of Na_2_S_2_O_4_ (2 mM, 5 mM, and 10 mM). Insets show higher magnification of the selected areas. **(B)** Representative phase-contrast images showing the morphology of MSFs after 24 h of treatment with different concentrations of atropine (30 μM and 300 μM). **(C)** Representative phase-contrast images showing the morphology of MSFs after 6 h of treatment with Na_2_S_2_O_4_ alone and Na_2_S_2_O_4_ combined with atropine. Insets show higher magnification of the selected areas. **(D)** Cell viability measured by CCK-8 assay at 6 and 12 h of Na_2_S_2_O_4_ treatment (*n* = 3 per group). **(E)** Quantitative analysis of cell viability at 6 h with various concentrations of Na_2_S_2_O_4_ (*n* = 3 per group). **(F)** Quantitative analysis of cell viability at 24 h with various concentrations of atropine (*n* = 3 per group). **(G)** Quantitative analysis of cell viability at 6 h in three groups (*n* = 6 per group). **(H)** Quantitative analysis of cell viability at 24 h in three groups (*n* = 6 per group). **(I)** Representative immunofluorescence images showing the cytoskeletal structure of MSFs stained for F-actin (red) and nuclei (DAPI, blue) in control, Na_2_S_2_O_4_-treated, and Na_2_S_2_O_4_ + atropine-treated groups. Insets show higher magnification of the selected areas, highlighting cytoskeletal disruption under the Na_2_S_2_O_4_ and protective effect of atropine on cytoskeletal fracture, analyzed 4 images per group. Data are presented as mean ± SEM. *P < 0.05, ***P < 0.001 compared to control; ††P < 0.01 compared to Na_2_S_2_O_4_. Scale bar: 200 μm **(A–C)** and 20 μm **(I)**.

To investigate the protective effects of atropine on MSFs under hypoxic conditions, we used a Na_2_S_2_O_4_-induced hypoxia model, a well-established method to simulate cellular hypoxia (Zhao et al., 2019; Sun et al., 2014). Phase-contrast microscopy images showed that Na_2_S_2_O_4_ treatment caused significant morphological changes, including cell shrinkage and detachment, indicative of reduced cell viability. In contrast, co-treatment with atropine mitigated these morphological changes, suggesting a protective effect of atropine against hypoxia-induced cellular damage (Figures 6A–C). Cell viability assays using CCK-8 confirmed that atropine significantly improved cell survival under hypoxic conditions induced by Na_2_S_2_O_4_ and the safety of up to 300 μM atropine to MSFs (Figures 6D–H). Furthermore, immunofluorescence staining of the cytoskeleton revealed that Na_2_S_2_O_4_ treatment disrupted F-actin filaments, whereas atropine preserved the integrity of the cytoskeleton, preventing the hypoxia-induced cytoskeletal disruption (Figure 6I). These results indicate that atropine can protect scleral fibroblasts from hypoxia-induced damage, potentially contributing to its overall protective effects in myopia.

### 3.5 Atropine modulates key signaling proteins in hypoxia-induced MSFs

**FIGURE 7:**
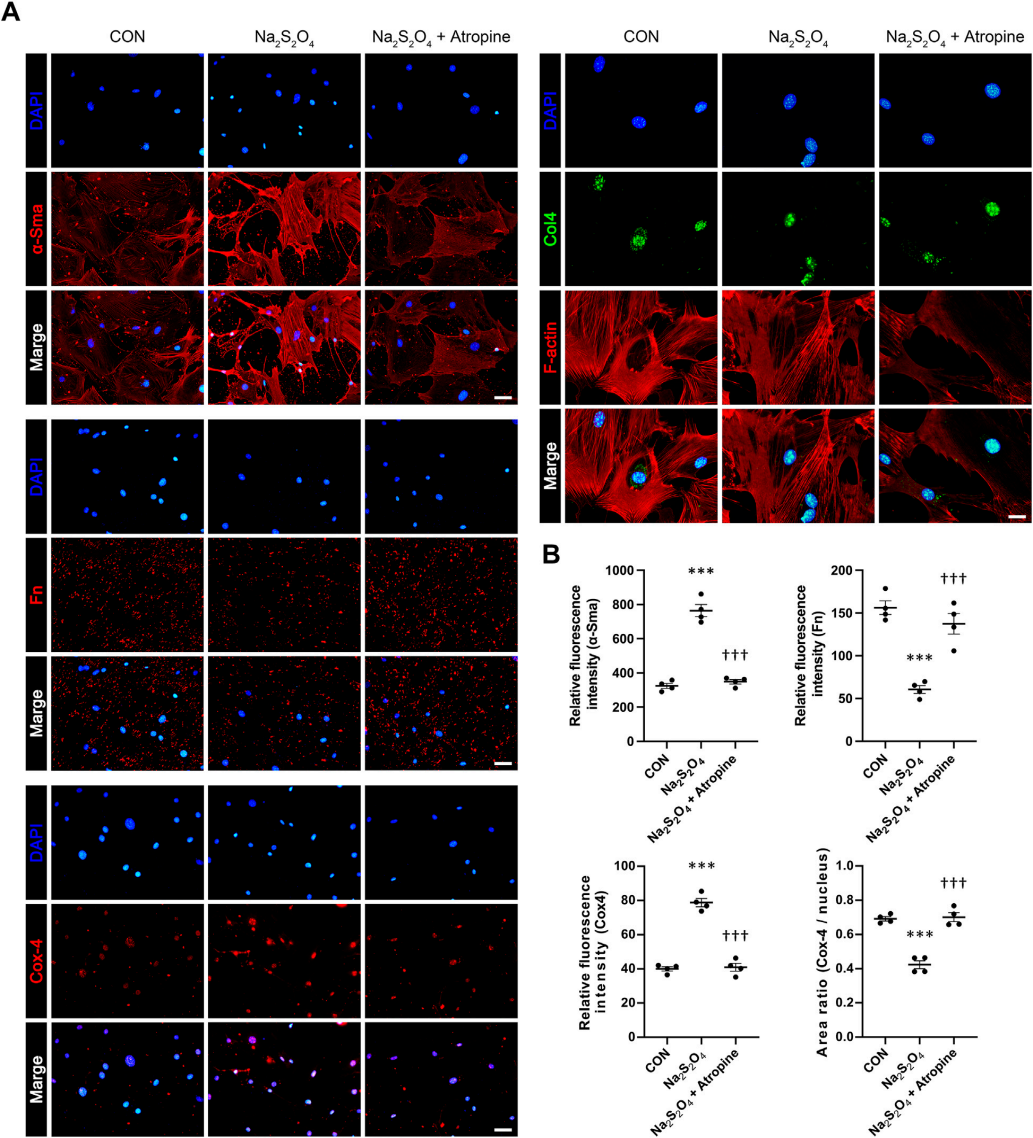
Effects of Atropine on the Expression of ECM Protein Expression in Na_2_S_2_O_4_-Induced Hypoxia Model in MSFs. **(A)** Representative immunofluorescence images showing the expression of α-Sma, Fn, Cox-4, F-actin (red), and Col4 (green) in control, Na_2_S_2_O_4_-treated, and Na_2_S_2_O_4_ + atropine-treated MSFs. DAPI (blue) was used to stain cell nuclei. **(B)** Quantitative analysis of relative fluorescence intensity of α-Sma, Fn, and Cox-4 in MSFs from the three groups, analyzed 4 images per group (*n* = 4 per group). The area ratio of Cox-4/nucleus is also presented. Data are presented as mean ± SEM. ^***^ *P* < 0.001 compared to control; ^†††^ *P* < 0.001 compared to Na_2_S_2_O_4_. Scale bars: 100 μm (α-Sma, Fn, and Cox-4) and 50 μm (Col4).

We further explored the effects of atropine on the expression of key markers associated with myofibroblast differentiation and ECM production in MSFs. Immunofluorescence analysis showed that Na_2_S_2_O_4_ treatment significantly increased the expression of α-Sma, a marker of myofibroblast differentiation, and decreased the expression of Fn and Col4, indicating weaken ECM production. However, co-treatment with atropine significantly reduced the expression levels of α-Sma and induced the expression levels of Fn and Col4, suggesting that atropine can inhibit hypoxia-induced myofibroblast differentiation and enhanced ECM production in scleral fibroblasts. The decreased ratio of Cox-4/nucleus indicated that Na_2_S_2_O_4_ inhibited mitochondrial function, this inhibitory effect could also be mitigated by atropine (Figures 7A, B). These results suggest that atropine can modulate cellular responses to hypoxia, thereby preventing pathological changes associated with myopia progression.

**FIGURE 8:**
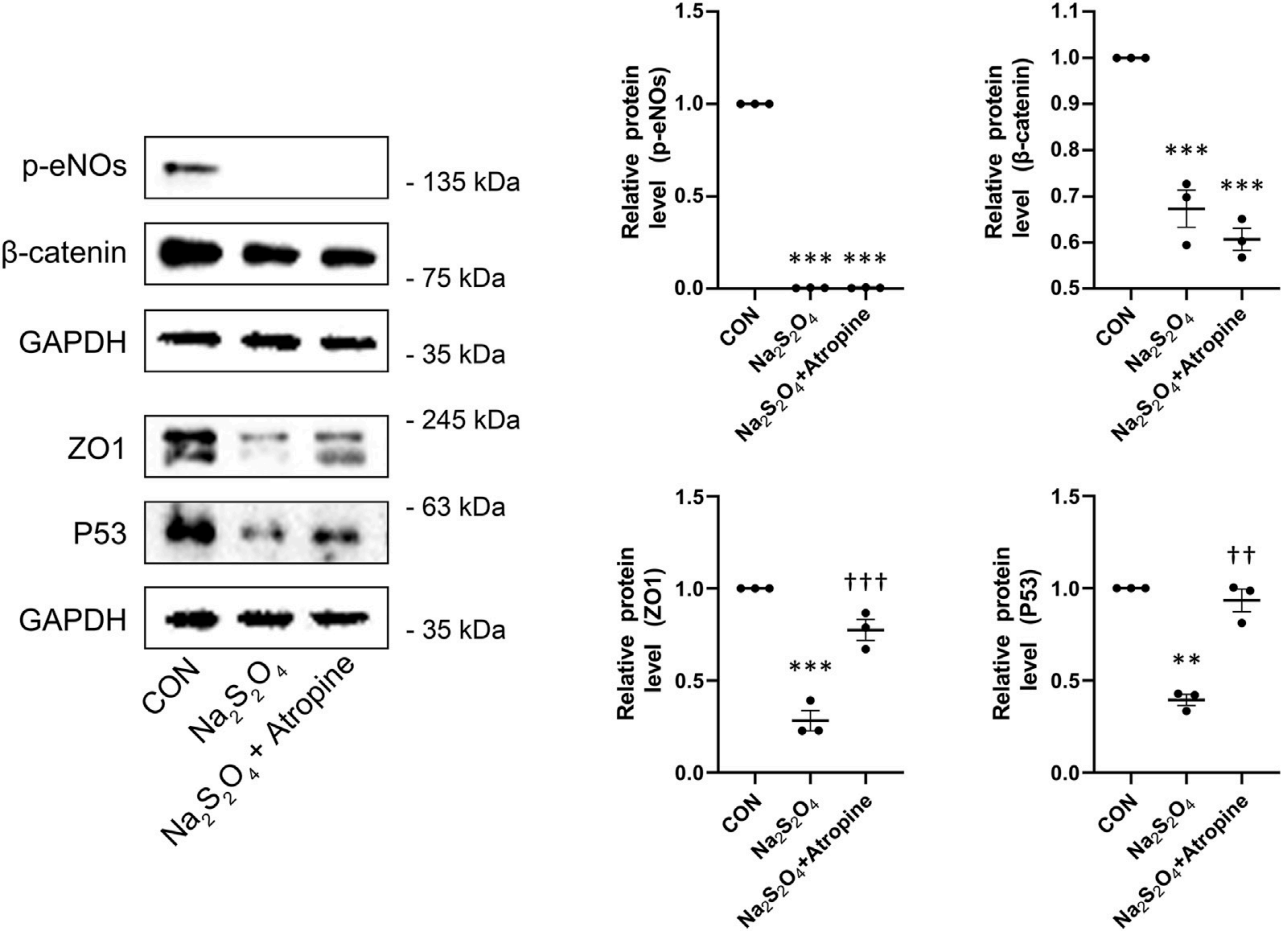
Effects of Atropine on Relative Protein Expression in Na_2_S_2_O_4_-Induced Hypoxia Model in MSFs. Western blot analysis showing the expression levels of p-eNOs, β-catenin, ZO1, and P53 in mock, Na_2_S_2_O_4_-treated, and Na_2_S_2_O_4_ + atropine-treated MSFs. The expression of each protein level was relative to GAPDH and was normalized to the control group (*n* = 3 per group). The right panels show the quantitative analysis of the relative protein levels of p-e-NOs, β-catenin, ZO1, and P53. Data are presented as mean ± SEM. ^**^ *P* < 0.01, ^***^ *P* < 0.001 compared to mock; ^††^ *P* < 0.01, ^†††^ *P* < 0.001 compared to Na_2_S_2_O_4_.

To elucidate the molecular mechanisms underlying atropine’s protective effects, we performed Western blot analysis to assess the expression levels of key signaling proteins in MSFs under hypoxic conditions. The results showed that Na_2_S_2_O_4_ treatment significantly decreased the expression of p-eNOs, β-catenin, and P53. In contrast, atropine co-treatment significantly induced the levels of P53 (Figure 8). Additionally, atropine increased the expression of ZO1, a tight junction protein, which was decreased by Na_2_S_2_O_4_ treatment, indicating that atropine can restore tight junction integrity under hypoxic conditions (Figure 8). These findings highlight the multifaceted role of atropine in modulating key pathways such as P53 signaling pathways and maintaining cellular homeostasis in response to hypoxic stress.

### 3.6 Complex PPI network shows potential pathways between MRs and myopia progression

**FIGURE 9:**
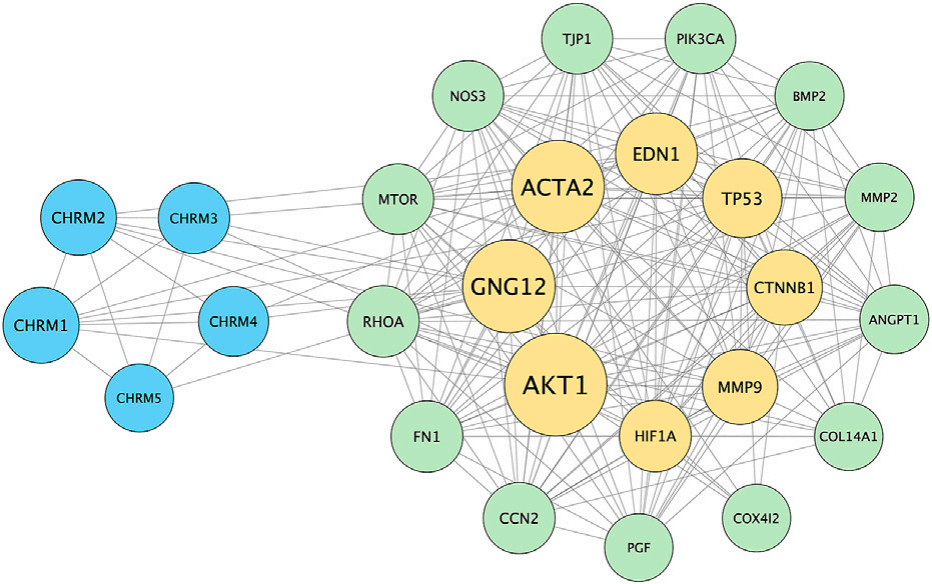
Protein-Protein Interaction (PPI) Network of Genes Associated with Myopia and Atropine Response. The PPI network was constructed using the STRING database to visualize interactions between muscarinic acetylcholine receptor (MR) subtypes (CHRM1-5) and various downstream effectors involved in ECM remodeling, choroidal blood flow regulation, and cellular hypoxia responses, which are relevant to the pathophysiology of myopia and the mechanism of atropine action. The network highlights key nodes such as AKT1, HIF1A, and CTNNB1 (β-catenin), indicating their central roles in mediating atropine’s effects on ocular tissues. These nodes are connected to MR subtypes, suggesting potential signaling pathways through which atropine might influence ECM stability, choroidal blood flow, and scleral function. This interaction network supports the hypothesis that atropine’s anti-myopia effects involve complex signaling cascades, warranting further research to validate these interactions and clarify the molecular mechanisms underlying atropine’s role in myopia control. Blue nodes represent muscarinic receptor subtypes, yellow nodes denote key signaling molecules and ECM components, and green nodes indicate other proteins involved in choroidal and scleral function. The edges represent protein-protein associations, including direct physical interactions and co-expression relationships.

The PPI network and visualization facilitated the identification of central nodes and pathways that may play critical roles in the therapeutic effects of atropine in myopia control, providing insights into potential molecular mechanisms for future investigation. The result illustrates key proteins such as AKT1, HIF-1α, and CTNNB1 (β-catenin) are central in the network, indicating their significant role in mediating the effects of atropine on ocular tissues. The interaction of these proteins with MR subtypes suggests a possible mechanism through which atropine may exert its effects, including regulation of ECM production and modulation of choroidal and scleral function. Additionally, the involvement of fibrosis-related markers like ACTA2 (α-SMA) highlights the potential pathways by which atropine could influence scleral stiffness and myopia progression (Figure 9).

## 4 Discussions

Our study comprehensively demonstrate that atropine not only significantly reduces axial elongation in FDM mouse models but also highlights the dual pathways through which atropine exerts its effects - by improving choroidal microcirculation and reducing scleral hypoxia. These findings provide novel insights into the multifaceted role of atropine in myopia control.

Atropine has the effect of relieving small blood vessel spasms and improving microcirculation, and most of the oxygen required by the sclera comes from the choroid. This characteristic of atropine suggests its potential to improve the hypoxic environment of the sclera and inhibit the scleral hypoxia pathway. On the other hand, atropine has shown the ability to directly target scleral fibroblasts and regulate their related biological functions. Therefore, we propose that atropine may control the progression of myopia through these dual pathways.

Our study demonstrates that atropine significantly reduces axial elongation in FDM mouse models. These results are consistent with recent findings that low-dose atropine can effectively slow myopia progression by targeting structural changes in the eye (Lee et al., 2024; Zhao F. et al., 2020). A systematic review and meta-analysis of 12 studies (*N* = 2,318 children) revealed that low-dose atropine (0.01%) exhibits minimal rebound effects after cessation, with a mean progression difference of −0.24 D/year compared to placebo. This contrasts sharply with higher concentrations (e.g., 0.5% atropine), which showed significant rebound progression (−0.68 D/year). The sustained efficacy and reduced rebound observed in this analysis support our hypothesis that low-dose atropine mitigates myopia progression through structural stabilization of the sclera and choroid, as demonstrated by our findings on ECM remodeling and hypoxia pathway inhibition (Lee et al., 2024).

Thinning of the sclera and choroid is important phenotype of myopia. In myopic eyes, scleral thinning mainly occurs in the region behind the equator, being most pronounced in the posterior pole. Among these areas, the peripapillary scleral border is the thinnest part, known as the zone of least resistance, forming the biomechanical anchor of the lamina cribrosa. Particularly in high myopia, the thickness of this area can be as thin as 50 μm, which may be a reason for the increased vulnerability of the optic disc and optic nerve in high myopia (Jonas et al., 2023). As the axial length of the eye increases, the thickness of the choroid decreases, most notably under the macular fovea, primarily affecting the medium and large choroidal vessels. Atropine treatment restored choroidal and scleral thickness in different regions of the eye, indicating its potential to counteract the thinning effects induced by myopia. Restoration of these structures is crucial for maintaining ocular integrity and function (Zadnik et al., 2023). Previous studies have shown that atropine enhances choroidal thickness, which plays a vital role in eye health by maintaining adequate blood flow and nutrient supply to the retina (Nickla and Wallman, 2010).

The choroid may regulate scleral growth in three ways to control axial length. First, in response to signals from the retinal pigment epithelium, the choroid may secrete growth factors that either stimulate or inhibit scleral growth, which is independent of choroidal thickness. Second, changes in choroidal thickness itself can affect the molecular signals reaching the sclera. This may be because the synthetic activity of the choroid is related to its thickness, or because an increase in choroidal thickness might create a greater barrier to signals from the retinal pigment epithelium, with a thicker choroid acting like a sponge to facilitate molecular entry into the sclera. Finally, the volume of the choroid may mechanically influence the thickness and volume of the sclera, thereby affecting the size of the eyeball (Nickla and Wallman, 2010). CVI is an important parameter for evaluating choroidal function. The increase in CVI observed in atropine-treated FDM mice suggests improved vascular health and blood flow within the choroid. This enhancement in CVI may help alleviate hypoxia-induced changes in myopia, as choroidal blood flow is critical for nutrient supply and waste removal in the retina (Sun et al., 2024). The improvement in choroidal blood flow could be a key mechanism through which atropine exerts its protective effects against myopia progression. Our correlation analysis revealed strong negative correlations between axial length and both choroidal and scleral thickness, emphasizing the interconnected growth and health of these tissues. This suggests that the therapeutic effects of atropine are not limited to a single ocular structure but involve multiple components of the eye, highlighting its multifaceted approach to controlling myopia progression (Ye et al., 2022; Xiong et al., 2020).

The modulation of ECM proteins, such as fibronectin and collagen, by atropine in the sclera of FDM mice indicates its regulatory effect on ECM remodeling. These findings suggest that atropine can induce ECM production, a key factor in myopia progression (Shi et al., 2024; Liu et al., 2023). This promotion of ECM production helps maintain the structural integrity of the sclera, preventing and counteracting excessive eye elongation (Shi et al., 2024; Chen et al., 2023). HIF-1α plays a key role in this process (Zhao C. et al., 2020). HIF-1 is universally present in human and mammalian cells. Under normoxic conditions, HIF-1 proteins synthesized are rapidly degraded via the oxygen-dependent ubiquitin-proteasome pathway. Only under hypoxic conditions can HIF-1 be stably expressed. HIF-1α, as a global regulator of the hypoxia response, activates the expression of hypoxia-responsive target genes, leading to a series of adaptive responses to hypoxia and maintaining oxygen homeostasis. Some researchers have found that prolonged HIF-1α signaling in chondrocytes leads to skeletal dysplasia by interfering with cellular bioenergetics and biosynthesis, reducing collagen synthesis, or causing excessive modification, which may also result in other ECM-related diseases, such as fibrosis (Stegen et al., 2019). Our study found that atropine significantly downregulated the expression levels of HIF-1α in the scleral and choroidal tissues of FDM mouse models, which is consistent with previous research findings (Kakinuma et al., 2005; Zhou et al., 2020).

The protective effects of atropine on MSFs under Na_2_S_2_O_4_-induced hypoxia were evident in our study. Atropine treatment mitigated significant morphological changes caused by hypoxia, such as cell shrinkage and detachment, indicative of reduced cell viability. Co-treatment with atropine preserved the integrity of the cytoskeleton, preventing hypoxia-induced cytoskeletal disruption, which suggests a protective effect against cellular damage in hypoxic conditions. This cytoprotective effect is crucial in maintaining the functionality of scleral fibroblasts, which play a pivotal role in scleral remodeling and myopia progression (Chen et al., 2024; Yuan et al., 2024).

To elucidate the molecular mechanisms underlying atropine’s protective effects, we assessed the expression levels of key signaling proteins in MSFs under hypoxic conditions. Na_2_S_2_O_4_ treatment significantly increased the expression of α-Sma, a marker of myofibroblast differentiation, and decreased Fn and Col4 levels, indicating weakened ECM production. However, co-treatment with atropine significantly reduced α-Sma expression and induced Fn and Col4 levels, suggesting that atropine can inhibit hypoxia-induced myofibroblast differentiation and enhance ECM production (Hu et al., 2021; Zhang et al., 2024). The decreased ratio of Cox-4/nucleus in Na_2_S_2_O_4_-treated cells indicated inhibited mitochondrial function, which was mitigated by atropine treatment. This finding underscores the role of atropine in maintaining mitochondrial integrity and function under hypoxic stress. Maintaining mitochondrial function is essential for the energy supply and metabolic processes required for cell survival and repair (Tripodi et al., 2021).

Na_2_S_2_O_4_ treatment significantly decreased the expression of p-eNOs, β-catenin, and P53, highlighting the activation of hypoxia and stress response pathways (Hernández-Bustabad et al., 2022; Attia et al., 2024; Lim et al., 2024). In contrast, atropine co-treatment increased the levels of P53 and ZO1, a tight junction protein, indicating that atropine can restore tight junction integrity and modulate key signaling pathways under hypoxic conditions. This ability to modulate multiple signaling pathways further supports the multifaceted role of atropine in protecting ocular health. Recent studies suggest that atropine’s effect on myopia progression may be mediated through its influence on the *Wnt*/*β-catenin* pathway, which are critical in regulating ECM remodeling and cell proliferation (Ma et al., 2014). Our findings open new avenues for understanding how atropine exerts its protective effects at the molecular level and provide a basis for further research into its potential combinatory use with other therapeutic agents.

The multi-faceted role of atropine in modulating structural, cellular, and molecular changes in myopia underscores its therapeutic potential. Atropine regulates the functions of the choroid and sclera by improving choroidal microcirculation and the hypoxic environment of the sclera, inhibiting the HIF-1α signaling pathway, and directly targeting scleral fibroblasts to inhibit ECM production and remodeling, thereby exerting its effect on controlling the progression of myopia. Despite the promising findings, our study has several limitations. The FDM mouse model may not fully replicate human myopia, and the long-term effects of atropine treatment were not assessed. Additionally, the molecular mechanisms underlying atropine’s protective effects require further investigation to fully elucidate its therapeutic potential. For example, evidence that atropine directly targets MRs on scleral fibroblasts to induce ECM production and remodeling, thereby exerting its myopia control effect, still requires further research. Nevertheless, we analyzed the potential associations between all subtypes of MRs and the factors relevant to this study. This PPI network supports the hypothesis that atropine’s anti-myopia effects may be mediated through complex signaling cascades involving MR subtypes, ECM components, and hypoxia-related pathways. Future studies are required to validate these interactions and further elucidate the molecular mechanisms underlying atropine’s role in myopia control.

While we acknowledge that HE staining has limitations, our results are consistent with prior findings and supported by complementary molecular data. Future studies could incorporate OCT or A/B scan for *in vivo* validation to further confirm the morphological changes observed in this study. These advanced imaging techniques would provide additional insights into the dynamic changes in axial length, choroidal thickness, and scleral thickness in live animals, complementing the histological data presented here. Na_2_S_2_O_4_ has been widely used to induce hypoxia in scleral fibroblasts and other cell types, it is important to acknowledge the limitations of chemical hypoxia models. Although our results demonstrate consistent hypoxia induction (e.g., HIF-1α upregulation and ECM remodeling), further validation using alternative methods, such as physical hypoxia (e.g., 1% O_2_) or genetic models, would strengthen the robustness of our findings. Future studies should explore these approaches to confirm the suitability of Na_2_S_2_O_4_ for hypoxia induction in MSFs and other ocular cell types.

In conclusion, atropine demonstrates significant potential in managing myopia progression by reducing axial elongation, restoring choroidal and scleral thickness, enhancing CVI, and protecting against hypoxia-induced damage. These findings, combined with robust clinical evidence from studies, which demonstrated that atropine effectively slows myopia progression in children with minimal side effects, strongly support the therapeutic application of atropine in clinical settings for myopia control.

## Data Availability

The raw data supporting the conclusions of this article will be made available by the authors, without undue reservation.
